# An Evaluation of the Potential of NMR Spectroscopy and Computational Modelling Methods to Inform Biopharmaceutical Formulations

**DOI:** 10.3390/pharmaceutics10040165

**Published:** 2018-09-21

**Authors:** Akash Pandya, Mark J. Howard, Mire Zloh, Paul A. Dalby

**Affiliations:** 1 Department of Biochemical Engineering, University College London, Gordon Street, London WC1E 7JE, UK; akash.pandya.15@ucl.ac.uk; 2 School of Chemistry, University of Leeds, Leeds LS2 9JT, UK; M.J.Howard@leeds.ac.uk; 3 Faculty of Pharmacy, University Business Academy, Trg mladenaca 5, 21000 Novi Sad, Serbia; zloh@live.co.uk; 4 NanoPuzzle Medicines Design, Business & Technology Centre, Bessemer Drive, Stevenage SG1 2DX, UK

**Keywords:** formulation, excipients, aggregation, NMR, molecular dynamics, molecular docking

## Abstract

Protein-based therapeutics are considered to be one of the most important classes of pharmaceuticals on the market. The growing need to prolong stability of high protein concentrations in liquid form has proven to be challenging. Therefore, significant effort is being made to design formulations which can enable the storage of these highly concentrated protein therapies for up to 2 years. Currently, the excipient selection approach involves empirical high-throughput screening, but does not reveal details on aggregation mechanisms or the molecular-level effects of the formulations under storage conditions. Computational modelling approaches have the potential to elucidate such mechanisms, and rapidly screen in silico prior to experimental testing. Nuclear Magnetic Resonance (NMR) spectroscopy can also provide complementary insights into excipient–protein interactions. This review will highlight the underpinning principles of molecular modelling and NMR spectroscopy. It will also discuss the advancements in the applications of computational and NMR approaches in investigating excipient–protein interactions.

## 1. Introduction

Protein-based therapeutics have become the leading class of pharmaceuticals on the market by sales. The most prominent are monoclonal antibodies (mAbs), which belong to the immunoglobulin family [1]. Currently, most of the monoclonal antibody therapies approved are formulated at protein concentrations of >30 mg/mL [2] and are administered parenterally as liquid injections (intravenous or sub-cutaneous routes) [3]. Due to instability in the gastrointestinal tract, capsular and tablet oral dosage forms have not yet been found to be a practical way to deliver such protein-based drugs [4,5]. Storage of biopharmaceuticals in the preferred liquid aqueous form must achieve a long shelf-life of up to 2 years at 4 °C to be approved [6]. However, the continuous threat of aggregate formation, precipitation, chemical degradation and other modifications has made designing the ideal formulation very challenging [7].

Aggregation is considered to be one of the most pressing challenges, and can occur at all stages of protein pharmaceutical development, manufacture, formulation and storage. The propensity of protein-based products to aggregate during various stages of manufacturing has also been reviewed thoroughly [8,9]. One strategy to address this problem is to understand the mechanisms that dictate aggregation pathways under relevant solution conditions. The currently known range of aggregation pathways and mechanisms have been reviewed extensively [10,11], and are not the focus of this review. Understanding detailed mechanisms is time-consuming and so the industry is typically under pressure to take an empirical approach to formulation, typically through combinatorial screening, to obtain products that are stable to aggregation, along with other degradation pathways. Such approaches often use convenient biophysical parameter determinations as indicators of aggregation propensity, such as the thermal transition midpoint (*T_m_*) for global conformational unfolding, or the temperature (*T*_agg_) at which aggregation is first detected. The capabilities and limitations of these rapid *T_m_* and *T*_agg_ measurements have been explored extensively, and have been found to correlate well with rates of aggregation at elevated temperatures [12,13]. However, these correlations disappear for aggregation kinetics under low-temperature storage conditions, because the aggregation mechanisms are no longer primarily driven by global protein unfolding [14,15,16,17].

Protein-based pharmaceuticals often require the addition of formulation excipients to ensure their stability. The major groups of excipients include: amino acids; buffering agents; sugars; osmolytes and surfactants. There is significant overlap between certain excipient groups, and many excipients carry out more than one function in formulations, including control of aggregation, vial surface adsorption, viscosity, shear-thinning, chemical instabilities, and container instabilities. Although pharmaceutical excipients are readily available, the mechanisms of action for each vary. A number of theories such as preferential interaction/hydration, volume exclusion/crowding, cation-π interactions, and electrostatic interactions, dispersive and aromatic interactions have been hypothesised [18,19,20].

High-throughput (HTP) methods have been used in the pharmaceutical industry for decades, primarily for identifying lead compounds in drug discovery, and several in depth reviews have focused on their applications [21,22,23]. Outlined below (Table 1) are some of the prominent high-throughput biophysical methods used to assist the excipient selection process for protein-based formulations. An extensive study on the use of HTP methods to design a formulation for a highly concentrated IgG mAb is reported here [24].

Given that high-throughput screening approaches are empirical, and hence time-consuming, and often only provide indirect surrogate evaluations of whether a protein is likely to be stable at low temperature over 2 years, there is a need to create a more fundamental understanding of aggregation mechanisms and the impact of different modes of formulation under these storage conditions. There is also the potential to use computational modelling approaches to both elucidate such mechanisms, and also to refine them to create better modelling methods.

**Figure 1:**
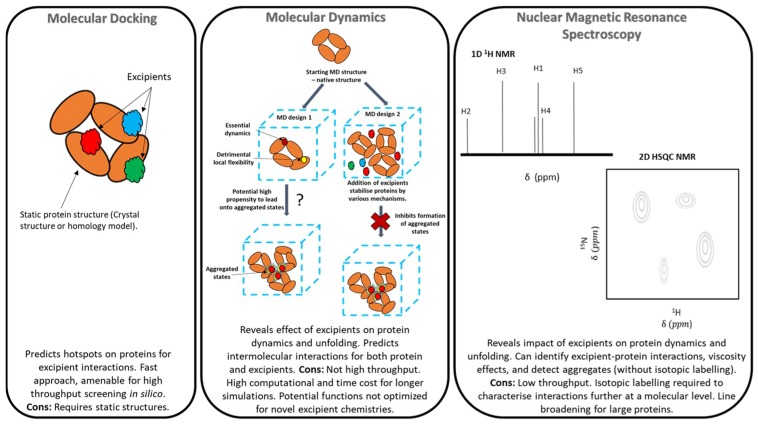
A schematic representation of the potential of in silico molecular docking, molecular dynamics simulation, and NMR spectroscopy to elucidate protein-excipient interactions to inform the rational design of protein-based formulations.

The combination of such approaches with more detailed biophysical analyses, such as NMR thus has significant potential to advance the understanding and predictability of formulation for biologics. A schematic shown in Figure 1 highlights the different capabilities and limitations of molecular modelling techniques and NMR. NMR spectroscopy provides structural and functional insights into molecules under interrogation through observations including the chemical shift and spectral line shape analysis that inform both on molecular structure in addition to intermolecular interactions. Such information has the potential to be utilized to validate and refine molecular modelling approaches designed to predict protein-protein and protein-excipient interactions, as well as the propensity of proteins to aggregate.

We start below with an informative summary of the underpinning principles of computational modelling and NMR spectroscopy. Based on these principles, this review reports on the complementary insights that can be obtained from Nuclear Magnetic Resonance (NMR) spectroscopy and computational modelling methods, to inform the rational design of protein-based formulations. We have a particular focus on the aggregation mechanisms observed under low-temperature storage conditions. These include those mechanisms driven by hot-spot interactions between proteins, and by local fluctuations within the native protein structure ensemble that can reveal aggregation-prone regions (APRs) [17,34,35,36,37,38]. Thus, it would also be useful to elucidate protein-excipient interactions with the potential to modify the ability of proteins to self-associate via hydrophobic hotspots or APRs on the surface, or by local fluctuations that reveal buried APRs.

## 2. Overview of Molecular Modelling, Methodologies and Limitations

### 2.1. Molecular Docking

**Figure 2:**
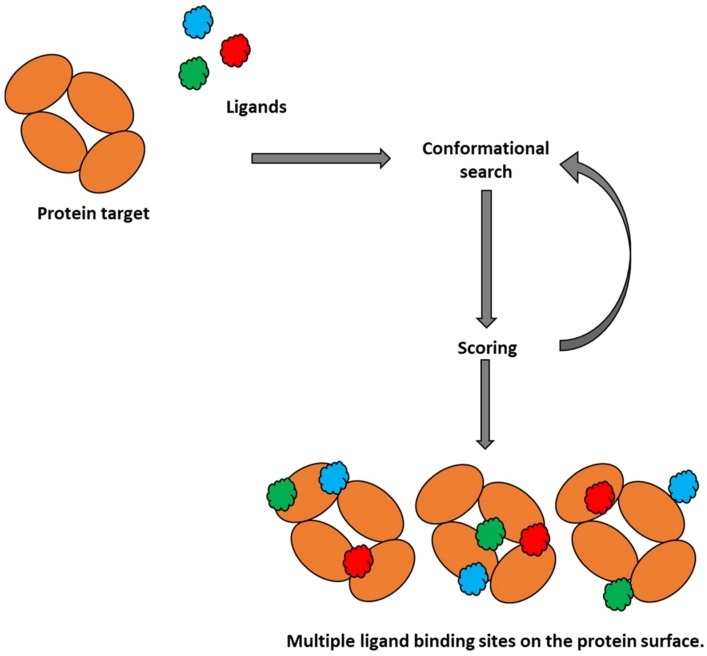
A schematic of the molecular docking process on a single multimeric protein.

Molecular docking is a computational chemistry tool that is extensively used in drug discovery processes [39], with more recent applications found in the drug development process. This method enables evaluation of intermolecular interactions between two molecules by predicting possible binding modes. The target molecule is a biopolymer, usually a protein, while the binding to a target can be predicted for either a small molecule (ligand-protein docking) as shown below in Figure 2 or another protein (protein-protein docking). The docking typically requires the known structure of the target molecule and seeks for an intermolecular complex with the most favourable binding pose based on the fit between target and its bound molecule, as well as the stabilization resulting from intermolecular interactions. Molecular docking generally involves generating a number of viable protein-ligand conformations by using search algorithms. Initially, a conformation of a complex (binding pose) is predicted in the form of the orientation of two molecules relative to each other. This is followed by estimation of the binding energies for each pose [40]. As absolute binding affinities are difficult to predict, scoring functions have been developed to establish a ranking of the predicted docked poses. This ranking of the generated conformations is achieved by using three main categories of scoring function. Force-field scoring functions use molecular mechanics and are established from calculations of atomic interactions including bond stretching or bending, torsional forces, van der Waals and electrostatic interactions [41]. Empirical scoring functions predict binding energies for docked conformations by considering van der Waals, hydrogen bonding, electrostatics and desolvation terms, whose relative weightings have been optimized through continual validation and refinement [42]. Lastly, knowledge-based scoring functions originate from experimental structural information [43]. The entropic effects contribution, an important component of protein-ligand binding energy, is usually taken into account through re-scoring or employing other methods [44]. A combined use of scoring and re-scoring allows selection of the most probable binding mode for a single ligand, or ranking of a series of ligands according to their predicted affinity for a selected target.

These scoring functions are usually implemented in software packages depending on their intended use, along with the appropriate conformational sampling methods that can also include different levels of molecular flexibility when generating binding poses. Rigid body docking allows for no conformational changes in the molecules of interest, and is thus the fastest. It is suitable for gaining insights into relative orientations of two docked proteins, or for scanning a protein surface with rigid small molecule ligands to identify binding hotspots. Notable docking programs that facilitate rigid protein-protein docking are Hex [45], and GRAMM-X [46]. These software packages sample protein-protein docked conformations based on a Fast Fourier Transform (FFT) algorithm. RosettaDock [47] is also used to predict the lowest energy docked protein-protein complexes by employing Monte Carlo approaches to position molecules in respect to each other, however it takes into account some flexibility by allowing the side-chains to move [47]. Protein-protein docking could provide possible insights into aggregation by identifying putative protein-protein interaction interfaces.

In contrast, flexible docking results in a conformational shift in the molecule of interest. This makes it favorable when studying ligand-protein complexes, and has the potential to predict and improve our understanding of specific interactions at the protein-ligand interface. The wealth of ligand-protein docking software packages available have increased over the decades. Some prominent flexible docking programs include AutoDock 4 [48], GOLD [49,50], and GEMDOCK [51]. The major limitation for molecular docking is the incorporation of protein flexibility. It is a well-known fact that upon ligand binding, a protein undergoes conformational changes. The flexible docking software packages mentioned above overlook ligand-induced binding effects and thus treat the protein as rigid [52]. However, there are software packages dedicated to flexible ligand-flexible protein docking that enable side-chain flexibility, including AutoDock Vina [53] and FlexX [54]. The protein-ligand docking may provide information not only on mode of binding within a specified active site, but it can provide information on possible interaction sites on the whole protein surface [55], which may allow evaluation of excipient binding on the protein surface (Figure 2).

The accuracy of docking predictions that do not account for protein flexibility and presence of other components the solutions (water, ions, buffers) have always been debated. A common solution is to set up molecular dynamics (MD) simulations that can potentially sample various protein conformations upon ligand binding in explicit presence of water molecules and buffers, where a most favourable docking pose can be used as a starting conformation for the simulation experiments.

### 2.2. Molecular Dynamics (MD)

**Figure 3:**
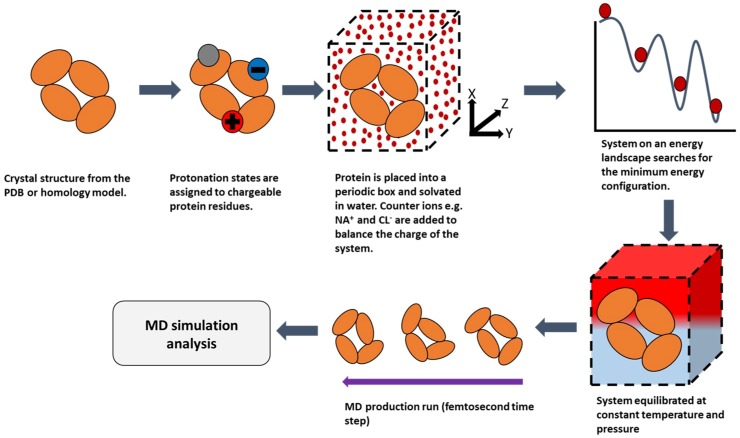
A schematic of the molecular dynamics process.

Molecular dynamics (MD) is a powerful computational modelling tool, which enables the following of subtle atomic motions of a system of interest as a function of time [56]. All-atom (classical) MD simulations sample configurations by integrating Newton’s law of motion to all the atoms in the system simultaneously over a femtosecond time step. A trajectory is recorded with precise atomic positions and velocities giving the user an indication of how the system evolves with time [57]. These positions, defined by Cartesian coordinates of all atoms in a system, allow calculation of the potential energy of the system and forces that act on each atom. Molecular dynamics commonly employs molecular mechanics approximations and use of the force fields, sets of equations and associated constants to reproduce geometries of molecular systems [58]. These are calculated based on the some commonly used force fields including CHARMM22 [59], CHARMM27 [60], AMBER [61] and GROMOS [62]. There is a wide array of software packages available, including but not limited to Gromacs [63], Amber [64], NAMD [65] and CHARMM [66], that have proven invaluable for the advancement of molecular dynamics. A general summary of the molecular dynamics process is provided below in Figure 3. A list of typically employed MD simulation times for various protein dynamics events are listed below in Table 2. The main limitation of MD is the small time-step, relative to a typical requirement for much longer simulation times. Longer simulations have been carried out on the millisecond time scale [67,68]. This may potentially place a strain on computational resources when simulating larger systems, however the benefits outweigh limitations due to the wealth of information that can be obtained about the system on the atomistic level [69].

## 3. Overview of Nuclear Magnetic Resonance (NMR) Spectroscopy

Nuclear Magnetic Resonance (NMR) spectroscopy detects nuclei of isotopes with spin angular momentum (e.g., ^1^H, ^15^N, ^13^C and ^19^F) in a magnetic field with the goal to provide analytical information regarding the structural and/or physical nature of any molecule under investigation. The resultant NMR spectra contain highly defined resonances (often referred to as peaks) that reflect the chemical nature of specific atoms in the molecule of interest. NMR has been applied in a diverse range of pharmaceutical applications that include, characterisation of proteins, drug discovery and design [70]. Despite the power and utility of NMR spectroscopy, linewidths of resonance peaks become larger as molecular weight increases. This due to a reduction in nuclear *T*_2_ relaxation times as the global tumbling of the molecule becomes slower. However, all is not lost as advances in NMR have allowed protein observations to be made over a wide-range of molecular timescales e.g., domain shifts (microseconds to milliseconds), side chain motions (picoseconds to nanoseconds) and changes in loop regions (nanoseconds to microseconds) [71,72].

For the purpose of this review, we are considering solution-state NMR methods where there are several observable NMR parameters including chemical shifts (δ) and relaxation times (*T*_1_ and *T*_2_). A chemical shift of a nucleus in a molecule arises due to a nuclear shielding or de-shielding effect of the NMR static applied magnetic field and is the result of electrons surrounding the nucleus. Chemical shifts (δ) are measured in parts per million (ppm) [73,74]. The power of chemical shifts provides structural information by distinguishing chemically-equivalent nuclei but where they are in different molecular environments. For example, the proton (^1^H) NMR of acetone (CH_3_COCH_3_) will only display one peak from two equivalent methyl groups but ethanol (CH_3_CH_2_OH) provides three ^1^H NMR peaks representing those in the methyl (CH_3_), methylene (CH_2_) and hydroxyl (OH) moieties respectively. These ethanol peaks are additionally subject to fine-structural splitting from spin-spin J-coupling, a concept taught to many undergraduates and found in NMR and organic chemistry textbooks. This example is a simplistic view of the power within chemical shifts and it cannot be stressed enough that structural and chemical environment influence NMR peak observations. For example, two alanine residues within a protein will both have methyl groups, but their observed chemical shifts will be dependent on their individual structural environments and so display diverse chemical shifts. Chemical shifts are ultimately derived from the Larmor frequency of resonance of each NMR peak, where the distance (in Hertz) between any two peaks is dependent also on the spectrometer magnetic field strength, because the Larmor frequency is proportional to magnetic field strength. When defining peak separation in terms of chemical shift, a 1 ppm separation for a proton (^1^H) spectrum on a 600 MHz (14.1 Tesla) spectrometer equates to a frequency separation of 600 Hz whereas for an 800 MHz (18.7 Tesla) spectrometer it would be 800 Hz. This field dependence of peak separation can be extremely useful when monitoring chemical exchange processes, as will be described further below.

NMR is a spectroscopic process that involves excitation and energy transfer between nuclear spin states which subsequently need to relax and return to equilibrium. This NMR relaxation is of fundamental interest to NMR spectroscopists as the time constants (*T*_1_ and *T*_2_) and rates (*R*_1_ and *R*_2_) of the associated mechanisms provide a wealth of information about the molecules being studied. Indeed, nuclear relaxation, and in particular relaxation dispersion, has found use as a tool to study protein folding and enzyme kinetics where chemical exchange events of interest, between species or molecular environments, can be quantified through their influence on NMR relaxation processes, as described in [75,76,77]. Each NMR-active nucleus in a molecule will possess a unique set of relaxation times from many fundamental molecular and bulk solution properties which is beyond this review. However, it is useful to note that molecular size and shape, proximity and the isotope of the nearest NMR-active nucleus, in addition to temperature, viscosity and the NMR magnetic field strength, all influence nuclear relaxation in quantifiable ways. The spin-lattice mechanism involves a nuclear spin exchanging energy with its surroundings (i.e., lattice), and thereafter returning to its ground state with time-constant *T*_1_ and relaxation rate *R*_1_. The second spin-spin relaxation mechanism involves the loss of phase coherence and subsequent loss of bulk magnetization that underpins the NMR signal, with time-constant *T*_2_ and relaxation rate *R*_2_. For both processes, *R_x_* = 1/*T_x_*. Although the processes giving *R*_1_ and *R*_2_ are independent events, the *R*_1_ and *R*_2_ relaxation rates converge for small molecules e.g., acetone and ethanol, whereas for larger species such as proteins in solution *R*_1_ and *R*_2_ are found to be significantly different. Furthermore, protein relaxation is extremely powerful and can differentiate the shape and internal motions of a molecule and reviews are available on this subject [78,79].

The linewidth of the individual NMR signals is proportional to *R*_2_ and therefore to the correlation (time of the molecule. The crucial point is that *R*_2_ has a proportionality to the correlation time (τ_c_); the time for a molecule to rotate through 1 radian (i.e., molecular motion) which is subsequently related to molecular size and shape. Assuming a sphere, correlation time can be estimated from [4πηr^3^/3k_b_T] where η—viscosity of the solvent, k_b_—Boltzmann constant, T—temperature and r the radius of the sphere. As a molecule becomes larger, r increases and so τ_c_ increases, which then makes *R*_2_ increase and is finally observed in NMR as an increase in linewidth. This is the fundamental reason why protein NMR spectra are broader than small molecule spectra. Increasing the temperature and/or lowering the viscosity would drop linewidth but many proteins are limited in their operational solvent conditions and thermal limits. Taking this concept of molecular size influencing NMR a step further, a ligand that binds to a protein would experience a significantly different correlation time, that is dictated by the size of the complex, compared to when the same molecule is free solution. In many cases, ligand binding is not permanent, but dynamic and is defined by an equilibrium with an affinity that can further influence the NMR observation [80].

When a molecule is in equilibrium between two or more states, the rate of exchange determines whether the chemical shifts of each of each state is visible or the chemical shift of a single time-averaged state is observed. The observation is thus dictated by the equilibria involved in exchange as well as the difference in nuclear spin relaxation rates *R*_1_ and *R*_2_. These two limits are known as slow and fast exchange in the chemical shift timescale but there is an intermediate condition where all resonances become broad and difficult to detect. At this condition, known as coalescence, the exchange rate constant is comparable to the difference (in Hz) between the Larmor frequencies of the two states. This ties in with the earlier concept of chemical shifts between peaks being frequency dependent and as the relationship between chemical shift and the equilibrium is not based on parts per million (ppm) but the fundamental Hertz distance between NMR resonances. Therefore, changing NMR field strengths can help move the observation between the slow, intermediate and fast exchange regimes.

### 3.1. Limitations

The potential for NMR spectra to become very challenging to interpret is very real when increasing the molecular weight of the protein. The number of proton resonances scales proportionally with molecular weight, but the spectral width remains constant, leading to increased crowding of peaks. In addition, line width increases with the molecular weight, leading to a further decrease in resolution and in signal intensity with the consequence that, resonance overlap increases rapidly with molecular size, until individual lines become too difficult to resolve. A commonly utilized workaround is to expand the NMR spectra from one to more dimensions (2D, 3D, 4D, etc.) to create more special representation of resonance peaks. Alternatively, data can be obtained at higher magnetic fields (e.g., at 14.1, 18.8, 21.1 and 23.5 Tesla that equates to 600, 800, 900 and 1000 MHz ^1^H resonant frequencies) to improve resolution. Other approaches include the addition of alternative NMR-active nuclei than ^1^H, such as ^15^N and/or ^13^C in addition to reducing spin-spin (*R*_2_) based relaxation by deuteration; the process of replacing ^1^H with ^2^H. As discussed, *R*_2_ is responsible for line broadening and reducing this process will create narrower lines. However, as discussed further below, TROSY NMR can be used to select slower NMR relaxation pathways to and therefore observe narrower lines, which then extends the range of molecular weight for which peaks can be resolved. Such a method is at its most powerful when combined with deuteration.

### 3.2. Protein-Observe Methods

As mentioned, the chemical shift is sensitive to the molecular environment around the nucleus and when a ligand or excipient interacts with a protein, physical characteristics for both are altered. The binding even creates a change in electron density and so influences the most prominent observable NMR parameter, chemical shift. As a result, chemical shift mapping (CSM) or chemical shift perturbation (CSP) methods are potential modes of investigation in protein-observe NMR [81]. CSM/CSP methods compare two protein NMR spectra, such as with and without the addition of a ligand, and track any changes in chemical shift and/or disappearance of peaks. These changes will identify any areas of the protein that are influenced by the binding event, with the largest changes being typically observed in the region around the binding site. In addition, careful experimental design can utilize a suite of NMR spectra, obtained over a range of ligand concentrations, to provide isotherms from which dissociation constants for the binding process can be determined.

**Figure 4:**
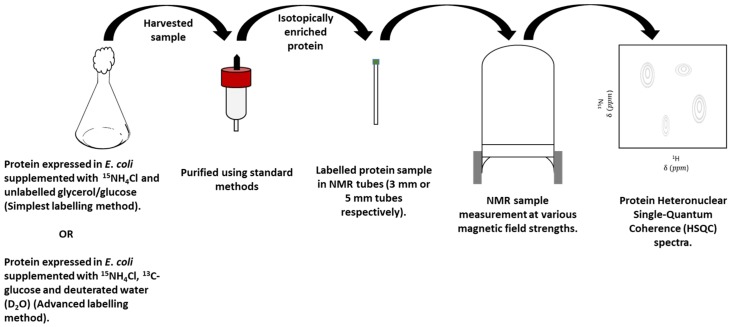
A schematic depicting the bacterial production of recombinant isotopically labelled protein, and recording of an NMR spectrum.

High molecular weight protein species such as whole mAb, Fab fragment and Fc region can be expressed with isotopic labelled nuclei, which enable greater resolution of peaks through the collection of multidimensional heteronuclear correlation spectra. There are several labelling protocols that exist for NMR studies. Uniform ^15^N isotopic enrichment is the simplest form of labelling a protein. The protein is expressed in *E*. *coli* (BL21) [82] grown on minimal growth media and supplemented by ^15^NH_4_Cl and unlabelled glycerol/glucose that is purified using standard methods to provide a +90% enriched protein product as shown in the schematic below in Figure 4. Heteronuclear single quantum coherence (HSQC) NMR spectra are 2D NMR experiments recorded to show all nitrogen-hydrogen correlations, which typically are dominated by backbone amide groups from the protein [83]. In the case of studying excipient–protein interactions, a series of HSQC NMR spectra can be recorded of the protein in presence and in the absence of the ligand. Binding effects of these interactions can be investigated by overlaying the series of ^15^N HSQC spectrums. If there is an interaction between the excipient and protein, the peaks on the spectra will be found to track from their initial position when no ligand was present [84].

More complex labelling approaches exist, such as ^2^H/^15^N/^13^C triple labelling which uses bacteria, yeast or insect cells as an expression system supplemented by ^15^NH_4_Cl, ^13^C-glucose and deuterated water (D_2_O). This form of labelling enables detailed mapping of whole structural changes in the protein upon excipient binding as it can offer significant coverage of the protein backbone and side chains. Deuteration replaces the aliphatic and aromatic protons for ^2^H across the protein to reduce the significant nuclear relaxation effect of ^1^H nuclei that facilitate line broadening. The proportion of ^1^H to ^2^H depends greatly on the carbon source used and whether H_2_O or D_2_O is present [85]. However, using specifically labelled methyl group probes (^13^CH_3_) in the study of high molecular weight proteins has proven useful in expanding the molecular weight limit reached with perdeuterated proteins [86]. Methyl groups tend to occur in the hydrophobic cores of proteins and provide an excellent probe for conformational modifications [87,88]. Several protocols for methyl group labelling are available [89,90,91,92].

**Figure 5:**
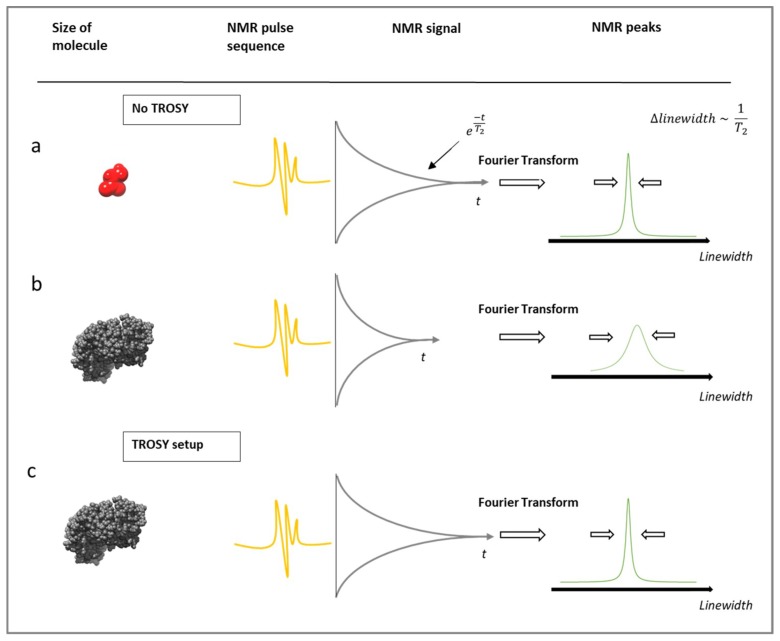
(**a**) The NMR spectrum for a small molecule ligand will depict a narrow linewidth due to a longer transverse relaxation time. (**b**) In contrast, a protein has a shorter transverse relaxation time and thus a broad linewidth is shown. (**c**) TROSY prolongs the transverse relaxation times and thereby improves the protein signal in the spectrum.

The increased relaxation already mentioned for proteins increases with increasing magnetic field strength, which creates additional issues. Consequently, several protein-NMR methodological advances have been utilized to address this challenge, the most popular being Transverse relaxation-optimised spectroscopy (TROSY) [93,94]. TROSY which uses spectroscopic means to reduce the observed *T*_2_ relaxation by selecting the slower relaxation pathway for observation. Remember, *T*_2_ is related to line width and accessing the slowest relaxing pathway will produce narrower resonance lines. Therefore, TROSY accompanied by various isotopic labelling techniques especially deuteration allows the study of biomolecules above 25–30 kDa [95]. The effect of TROSY on the relaxation rate of an excipients and proteins is demonstrated in Figure 5.

TROSY, when combined with higher magnetic fields (e.g., equal to or greater than 18.8 T–800 MHz ^1^H) and the introduction of cryogenic probes has enabled NMR fingerprinting using ^1^H/^2^H, ^15^N ^13^C isotopes at natural abundance of full mAb, Fab, Fc and other therapeutic proteins possible [96,97,98,99].

### 3.3. Ligand-Observe Methods

Ligand observe methods exploit the dependence of relaxation rates on molecular size described earlier in this review, specifically via the difference in molecular weight between a small ligand and a significantly larger protein. Nuclear Overhauser Effects (NOEs) are defined as the change in intensity of NMR resonances caused by dipole-dipole coupling. The sign and the magnitude of the NOE is dictated by the hydrodynamic radius (r^6^) and the correlation time (τ_c_). This makes NOEs available to detect intramolecular and intermolecular interactions. Large molecules tend to tumble slower and so it is anticipated that a negative NOE will be observed. In contrast ligands tumble fast resulting in a positive NOE [100]. Two prominent NOE based NMR methods include Saturation Transfer Difference (STD) and WaterLOGSY (Water Ligand-Observed via Gradient spectroscopy.

**Figure 6:**
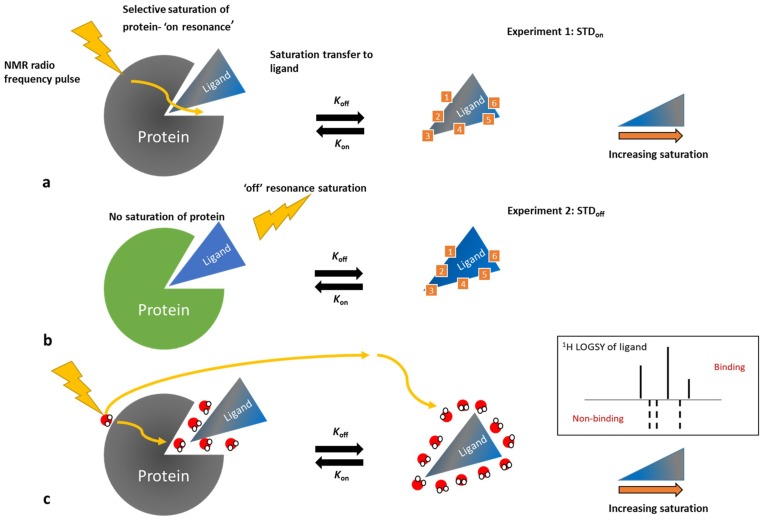
Schematic representation of saturation transfer difference (STD) NMR (**a**,**b**) adapted from [101] and WaterLOGSY (**c**) NMR. Increasing saturation of the ligand’s resonances is indicated by a colour gradient from blue (no saturation) to grey (high saturation).

STD NMR involves recording a STD_on_ spectrum, whereby only the ^1^H protons of a protein and not the excipient (ligand) are selectively saturated by a narrow selective ^1^H NMR pulse typically placed between −1 and −3 ppm. The saturation is transferred throughout the protein via spin diffusion and onto any bound ligand, resulting in a reduction in the intensity of the ligand resonances as shown in Figure 6a. In order to detect this transfer, the ligand must dissociate from the protein. Therefore, the dissociation constant (*K*_D_), which describes the affinity of an excipient to the protein, has to be favourable for this process. A favourable *K*_D_ also allows multiple excipient molecules to bind and dissociate, each receiving saturation from the protein and increasing the observable signal in the STD_on_ experiment, as compared to when a small number of molecules bind during saturation. STD requires a difference spectrum which is created by subtracting a second control experiment spectrum (STD_off_) acquired when no saturation of the protein takes place and is usually created by saturating outside the protein chemical shift envelope. In reality these two experiments (STD_on_ and STD_off_) are acquired in a single interleaved experiment and processed simultaneously using a spectrometer macro to provide both 1D datasets STD_off_ and STD_diff_ (a spectrum automatically created as the subtraction of STD_off_ and STD_on_). The nature of the transfer from protein to ligand uses a negative NOE which manifests for any peaks from nuclei involved in saturation transfer having lower magnitude signals in the a STD_on_ spectrum when compared to the STD_off_ spectrum. Any nuclei not involved in saturation transfer will have resonances of identical magnitude in both STD_off_ and STD_on_. The resulting difference spectrum between STD_off_ and STD_on_ will display only resonances where saturation transfer has occurred [101]. Additionally, and with careful experimental set-up, the magnitude of the difference spectrum also provides an indication of the orientation of the molecule upon binding to the protein.

WaterLOGSY [102,103] operates by observing a NOE between the ligand and water molecules. The water molecules are either in the bulk solution or in the vicinity of a protein’s surface. In the case of the latter, the water molecules take up the tumbling characteristics of the protein, resulting in a negative NOE upon ligand binding. Conversely, the ligand molecules interacting with the bulk water will result in positive NOE, by inheriting the tumbling characteristics of the water molecules. Binders and non-binders can be identified via a spectrum displaying negative and/or positive peaks. Control experiments with and without the protein are required to ascertain the sign of the positive NOE (Figure 6c).

Diffusion based experiments such as pulsed-field gradient (PFG)-based pulse sequence are another group of ligand-observe experiments. The PFG experiments enable the study the general molecular displacements occurring in either complex or simple mixtures. Diffusion measurements can be obtained by experiments using either spin echo (SE) or stimulated-echo (STE). When combined with the acronym PFG, the full abbreviation becomes PFGSE and PFGSTE, respectively. Diffusion data can be demonstrated in many ways as diffusion curves or 2D maps. A pictorial representation of a DOSY spectrum as a 2D map is common, with one dimension constituting to chemical shifts and the other dimension representing diffusion coefficients. In addition to identifying the various components of a mixture, DOSY can also offer an insight into the hydrodynamics of the molecular system of interest by observing the self-diffusion of molecules in solution [104]. DOSY works by utilizing pulsed-field gradient NMR spectroscopy to measure translational diffusion of molecules where the pulsed-field gradient can spatially label resonances in a molecule of interest.

If 2D DOSY maps are used, they should be accompanied with diffusion curves that display the NMR peak intensity as the NMR gradients are perturbed. This curve can be analysed to obtain a diffusion constant. It is also important to define the process of DOSY calibration using known diffusion standards such as dioxane. PFG experiments can provide insights into mixtures containing excipients and these will be highlighted in the next section.

## 4. Nuclear Magnetic Resonance (NMR) Spectroscopy Applications in Aggregation and Formulation

Here we discuss the applications of NMR already implemented in the study of aggregation and formulation design. We also discuss the significance of the examples provided. A key component of many protein aggregation mechanisms is the formation of oligomeric protein complexes that are thermodynamically unfavourable, and therefore exist only transiently and at very low populations. Heteronuclear spin relaxation rates have been measured previously to determine weak association constants for the transient formation of oligomers of bovine low molecular weight protein tyrosine phosphatase (BPTP) under equilibrium conditions [105]. The approach combined hydrodynamic calculations with the conventional measurement of *R*_1_ (longitudinal) and *R*_2_ (transverse) at different BPTP concentrations, to reveal the formation of tetramers, and also that the tetramerisation interface was formed by a cluster of residues on the surface of the dimer. However, in irreversible protein aggregation mechanisms the soluble oligomers form under pseudo-equilibrium as transient and rarely populated intermediates, and so their detection by NMR is far more challenging. A significant amount of work using NMR has been focused on amyloid fibril aggregates which play a huge in role several prominent diseases such as Parkinson’s and Alzheimer’s. Fibril formation of α-synuclein (αSyn), responsible for Parkinson’s disease has been studied using Paramagnetic Relaxation Enhancement (PRE) NMR to depict the various contacts between heterogeneous disordered monomers. PRE NMR involves a nitroxide spin-label being attached to a particular protein region and while in its oxidized state (paramagnetic) improves the relaxation during the ^1^H-^15^N HSQC experiment [106]. PRE-based contacts exceed the conventional NOE distances (≤5 Å) by at least 4-fold and provide probes that interrogate molecular structure over long-ranges [107].

The exchange dynamics between amyloid-β (Aβ) monomers and polydisperse, NMR-invisible (‘dark’) protofibrils was investigated by a novel technique called Dark-state Exchange Saturation Transfer (DEST). DEST follows similar principles to that of Saturation Transfer Difference (STD) NMR. This structural and kinetic study of the protofibril formation was highly significant as the build-up of toxic, soluble aggregate forms of Aβ, then forms larger assemblies which contribute to the development of Alzheimer’s disease. The main findings showed ^15^N-*R*_2_ values being significantly larger for Aβ42 than the closely related variant Aβ40. This supported the known observation that Aβ42 demonstrated a higher propensity for rapid aggregation and fibril formation than Aβ40 [108].

The human immunoglobulin kIV light chain variable domain (LEN) has the potential to be converted into amyloid under stress conditions. CPMG [109,110] relaxation NMR experiments were used to identify residues undergoing slow millisecond motions. Multidimensional solution NMR experiments were implemented at physiological and acidic pH. The main findings revealed that certain flexible residues at the dimer interface drive the formation of partially misfolded conformers. By identifying the specific residues and regions which contribute to the early stages of unfolding, such work may pave the way for the rational design of stable tertiary and even quaternary structures, that prevent aggregation [111].

NMR spectroscopy considers the structural properties of therapeutic proteins, however overlook the presence of a solvent, typically water. The solvent plays a vital role in influencing protein dynamics. A new method called water proton NMR was proposed to use the transverse relaxation (*T*_2_) time of water protons to quantify protein aggregation. BSA (66 kDa) and γ-globulin (150 kDa) were subjected to temperature-induced aggregation. The *T*_2_ of water protons increased linearly with the percentage of aggregate formation. The correlation was consistent at high and low magnetic fields [112]. Furthermore, a pH-induced aggregation procedure was also implemented on human insulin. A non-linear trend between *T*_2_ of water protons and aggregates was observed in this case [113]. The water NMR method was extended to investigate mAb aggregation under various stress conditions, in which the transverse relaxation of water protons detected aggregate formation [114]. The NMR technique itself provides a rapid and non-invasive method in characterising the extent of aggregation in finished products.

A combined approach using DOSY-NMR and DLS was used to measure diffusion coefficients and particle size distributions for five commercially available insulin drug products. The authors revealed that DLS was more effective in detecting larger aggregates than DOSY-NMR due to the higher sensitivity to high molecular weight species. In contrast DOSY-NMR was found to be more suitable in detailing excipient behaviour in the formulation [115]. The findings do not diminish the potential of NMR in the study of aggregates, but rather emphasize the importance in selecting the appropriate NMR method based on prior information about the system being investigated.

There has been a significant contribution towards characterising solid dosage form formulations with techniques such as ^15^N Dynamic Nuclear Polarisation [116] and ^13^C Magic angle spinning NMR [117,118,119]. However, the applications of NMR on solid dosage formulations is beyond the scope of this review.

Antibody-based therapeutics are frequently formulated with small concentrations of sugars, amino acids, buffer salts and polysorbates. The presence of such excipients may potentially induce protein structural changes which solution NMR can pin-point. Several notable NMR studies have been carried out to assess the quality of new-age bio-therapeutics. Panjawani et al. have implemented 2D NMR fingerprinting assays to detect the effects of excipients and pH conditions on the conformations of two interferon (IFN) proteins (α-2a and α-2b). NMR spectra were recorded for both proteins and compared to reference spectra already recorded by regulatory agencies. The first stage of the formulation process was to add excipients used in products such as Roferon-A^®^ and Intron-A^®^. A deformulation process using Cation exchange chromatography was implemented to analyse the various components of the formulation. A series of 2D HSQC NMR spectra were recorded following the formulation and deformulation process under various pH conditions. The study revealed that there was no alteration of the IFN structure during the deformulation process. Below pH 3, the protein was found to unfold and at pH 4.5 the NMR spectra showed a tendency of the protein to oligomerise even though the tertiary structure was intact [120]. Similar NMR methods have been implemented on formulations relating to recombinant methionyl human GCSF (Neupogen^®^ by Amgen) in preparation for subsequent biosimilars entry into the market [121] and also on antibodies in presence of common excipients, Tween^®^ [122]. The three studies highlighted above all demonstrate the potential of NMR to guide the formulation process by providing structural insights into changes in the active ingredient under various formulation conditions.

The NMR methods for formulation design so far discussed all involve a form of isotopic labelling. However, Golovanov and co-workers have attempted to address the challenge of working directly with industrial formulation samples, by using 1D ^1^H NMR methods. One dimensional NMR spectra were recorded for mAbs in presence of an excipient mixture arginine glutamate. The ^1^H NMR experiments helped to identify conditions whereby protein-protein interactions were restricted. They also revealed that translational diffusion measurements were less useful than transverse relaxation data in finding the most suited formulation [123]. The other ligand-based approach mentioned earlier was diffusion NMR, this technique was used to differentiate NMR signals of the excipient that may potentially mask mAb and insulin NMR signals [124,125,126]. Although, 1D ^1^H NMR spectra are unsuitable for in-depth structural understanding of proteins, they can still provide an initial insight into whether the protein of interest is in its folded or unfolded state within formulations. 

## 5. Molecular Modelling Applications in Aggregation and Formulation Design

Ligand-protein and protein-protein interaction sites are functionally important for modulating protein stability, including potentially for the control of aggregation in therapeutic protein formulations. Steering the design of such sites can be challenging as often there are large surface areas involved, structural dynamics and the presence of solvent molecules [127,128]. Strategies to engineer these sites include mutating residues that are involved in the ligand-protein and protein-protein interactions. Although there are a significant number of available protein and peptide structures obtained by NMR or X-crystallography (PDB), there is a lack of experimental information on protein-protein interfaces, particularly those involved in protein aggregation. MD has been found to be a useful tool in predicting the influences of various factors on protein structures, including guiding site-directed mutagenesis [129].

Protein unfolding may lead to aggregated states, which itself presents as an immense challenge in maintaining conformational stability. Daggett and Levitt have simulated unfolding pathways using MD to understand the mechanisms behind protein folding [130]. MD has also been found to be crucial in providing molecular insights into the amyloidogenesis process. Simulations at neutral and lower pH conditions were carried out to characterise the conformational changes of a prion protein from its native cellular isoform (PrP^C^) to an infectious form (PrP^Sc^). The latter is known to lead to neurodegenerative diseases. The protein structure was found to be intact at a neutral pH, whereas at lower pH there was more flexibility in the structure. Furthermore, the total sheet-like structure increased via the native β-sheet and an additional portion formed in the N terminal of PrP [131,132].

Daggett and co-workers hypothesized that rarely formed α-sheet structures are shared by amyloidogenic proteins which are linked to toxicity. Molecular Dynamics was used to generate structures for the rational design of novel anti-α-sheet peptides which were also tested experimentally. These α-sheet structures provide a suitable target for neutralising the toxicity and preventing fibril formation [133]. The novel α sheet design has the potential to prevent aggregation in several amyloid proteins Aβ Alzheimer’s and amylin (type 2 diabetes) as they bind to the toxic species [134].

An extensive review of the wealth of computational tools to determine aggregation-prone hotspots has already been carried out. The review highlights specific regions in either the structure or in the sequence of a protein that induces aggregation. It proposes a list of sequence based computational methods whereby Aggregation Prone Regions (APRs) can be identified [135]. Some of the more prominent tools include; AGGRESCAN [136], TANGO [137] and PASTA [138]. A structural tool to identify APRs has been used on therapeutically relevant mAbs. The Spatial Aggregation Propensity (SAP) method employs molecular dynamics to map fluctuations and identify the number of aggregation prone hydrophobic regions exposed on the antibody surface. These hydrophobic patches can be exposed natively, via fluctuations or conformational shifts and can be observed by molecular simulations. A high SAP value meant that there was a presence of APRs and these were used to inform of potential mutations that inherently improved antibody stability [139,140,141].

Computational simulations can potentially provide molecular insights into drug formulations. Although small molecule formulations are beyond the scope of this review, there are two notable modelling studies that employ docking and MD to study their respective formulations. A combined approach using both methods was used to investigate the interactions between hydrophilic excipients; lactose, hydroxypropyl methyl cellulose (HPMC), mannitol and the poorly soluble, Bicalutamide (BIC). BIC is a non-steroidal antiandrogen drug used to treat prostate cancer. A Lamarckian genetic algorithm within the AutoDock 4 software package [48] was used to seek out the lowest binding energy of BIC-excipient during docking. The best conformation of each BIC-excipient complex was then used as the starting MD structure. MD simulations of the docked complexes revealed Lennard-Jones interactions between BIC-HMPC/mannitol and coulomb interactions between BIC-lactose. Lactose formed the most hydrogen bonds with water and provided the best dissolution performance in experimental studies [142]. Jha and Larson conducted a detailed molecular dynamics study to assess the effects of polymeric excipients on a Phenytoin, which exhibits poor solubility in water. This work demonstrates the use of MD utilities such as radial distribution functions between API-API molecules to characterise aggregation and also between excipient-API to flesh out potential interactions [143].

As mentioned earlier, excipients interact with proteins via many different mechanisms. Arginine is particular has several mechanisms of actions, which makes it fascinating to study by MD. A series of aqueous molecular simulations to investigate arginine’s aggregation inhibition properties were set up. The simulations revealed arginine molecules had the tendency to form hydrogen bonded clusters when in solution. The hydrogen bonds were found to be stronger within clusters than those between arginine and water. A similar cluster formation was observed upon addition of proteins. The clusters effectively crowded out protein-protein interactions. By contrast, cation-π interactions between arginine and the protein were found to stabilise the unfolded intermediates [144].

In order for a protein to be conformationally stable, it would have to demonstrate good solubility in water. An excipient mixture of arginine (l-Arg) and glutamate (l-Glu) has been explored experimentally as a way forward to inhibit aggregation in proteins [145,146]. Preferential interaction coefficients were derived in simulations to investigate this behaviour further. Preferential interaction coefficients are a measure of the excess number of excipient molecules in the vicinity of a protein compared to that in the bulk solution. The simulation conditions included a combination of Arg-Glu mixtures at a range of concentrations, as well as single component l-arginine and l-glutamate system in the presence of the well-characterised Drosophila Su dx protein WW34. The main findings indicated that the equimolar mixture system enhanced the protein solubility more than systems consisting of single co-solvent components. Again, the increase in crowding and hydrogen-bond formation between co-solvent molecules led to a suppression of protein-protein interactions [147]. Another, MD study investigated Arginine’s role as an eluent during Protein A chromatography. The main purpose of this complementary study was to witness the effects of adding an excipient in an attempt to limit aggregate formation at a crucial manufacturing step. Addition of arginine was successful in disrupting the affinity between Protein A and an antibody. Meanwhile, another excipient, citrate, was found to reverse the process [148].

**Figure 7:**
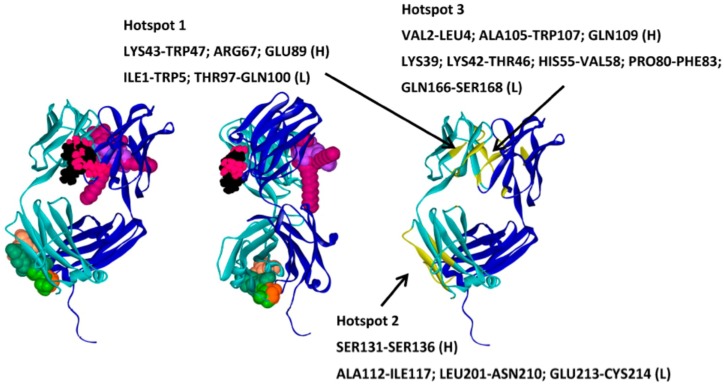
Binding hotspots for eight commercial excipients on the A33Fab surface. Reprinted from [156] with permission.

Similarly, MD simulations have been carried out with other excipients such as polyethylene glycol (PEG) [149], polyvinyl alcohol [150], trehalose and its derivatives [151,152,153]. Mechanistic insights into excipient interactions has also been studied using molecular dynamics in freeze-dried systems [154,155]. A recent computational study has implemented molecular docking approaches to identify binding hotspots on a Fab A33 fragment surface, for a set of commercially available excipients as shown below in Figure 7. The excipients selected for the study were from various categories such as amino acids, sugars, surfactants and osmolytes. Each excipient pose was characterized in terms of their predicted in silico binding energy, and also the Fab residues with which they interacted. All eight excipients were found to bind on three particular hotspots. Protein-protein docking was implemented on two Fab A33 molecules using Hex [45] and Grammx [46]. This allowed an appraisal of the interacting Fab residues with excipients and whether they coincided with protein-protein interaction sites. The regions through which two Fab molecules are predicted by docking to interact during aggregation, can potentially be shielded by adding excipients that also bind to those regions. The presence of such an effect was validated with the experimental determination of thermal stability for each formulation [156].

There have been efforts to demonstrate the possibility of using non-conventional classes of excipients such as anti-inflammatory drugs to enhance therapeutic protein stability. For example, the anti-inflammatory drug Dexamethasone phosphate was docked onto an in silico-built model of the Bevacizumab^®^–Bevacizumab^®^ interface to identify potential binding sites. A region on the Fc structure was found to interact with a Fab fragment on the second bevacizumab molecule. An interaction with Dexamethasone phosphate and Lys 445 on the protein was revealed to potentially suppress dimerization. Bevacizumab^®^ is a humanised whole mAb that is used to treat various cancers and eye disease [157,158].

## 6. Future Perspectives

Molecular modelling and NMR spectroscopy are promising techniques for improving the understanding of the structure and dynamics of proteins and the impact of formulation excipients. However, the current wealth of literature only demonstrates individual use of these techniques in the study of aggregation and designing formulations. There are opportunities to fill this void by combining computational and NMR methods. Molecular Dynamics and NMR share a complementary relationship, whereby observable NMR parameters can be used to validate those derived by molecular simulations. This has been made possible by the introduction of methodologies such as CamShift [159,160], which can be used to evaluate molecular dynamics derived backbone chemical shifts of proteins. The significance of this going forward may help the characterisation of protein motions on an atomic level, which ultimately can be invaluable when studying aggregation and informing the design of formulations.

Molecular docking has proven to be effective in finding excipient-binding hotspots on a protein’s surface. During drug discovery, 2D ^1^H-^15^N HSQC chemical shift perturbations (CSPs) have been used to guide molecular docking by selection of binding sites [161]. This also provides an excellent way of refinement and/or validation of molecular docking of excipients where low affinity binding and possibly non-specific interactions may occur. Once binding site(s) and results from docking are experimentally validated for a range of excipients by NMR, molecular docking would then have the potential to become more predictive, and therefore adopted in formulation design. Excipients could be screened in silico against validated protein binding-sites for local structure stabilisation, or for the shielding of protein surfaces that otherwise self-interact. Potentially even the design of novel excipients may be undertaken. Eventually, as accuracy of docking improves, this could be converted into an in silico high-throughput process which provides detailed insights into the nature of excipient–protein interactions.

Such computer aided formulation design can be complemented with molecular dynamics simulations by taking into consideration the simultaneous presence of multiple components, by including not only water, salts and buffers, but also including a combination of excipients. This would then enable a more detailed understanding of the complex interactions found between the effects of excipients, for example through competing affinity for either the same binding site or multiple binding sites. Ligand observe NMR methods may be used to confirm these in silico results. Judicious analyses of trajectories may also indicate a possible rationale for the effects of excipient combinations on protein structure and/or stability.

The computer modelling approaches may have higher significance during the development stages, particularly in industry where the availability of protein (native or isotope enriched) is low, and not yet fully characterized by NMR. As the protein production and purification processes are established, the issue of the availability for both NMR and experimental formulation, becomes less important. At those stages, an extensive set of experimental information may be acquired to inform the rational design of formulations, but will be able to build upon a wide pre-screen of available excipients in silico.

## 7. Conclusions

This review has highlighted the principles that govern molecular modelling tools and NMR spectroscopy. It also has given an overview of the applications of NMR and molecular simulations to provide atomic level insights into possible interactions between therapeutic proteins and excipients. A key aim going forward will be to harness the relationship between NMR and computational modelling, and the insights they provide, in the design of new formulations that will ensure product stability.

## Figures and Tables

**Table 1:** High throughput biophysical methods used for excipient screening.

Biophysical Method	Details	Limitations	Application References
Raman spectroscopy	Measures shifts in energy (wavelength) of photons re-emitted after interaction with molecular vibrational modes. Provides an empirical signature of protein structure, that can be used to monitor changes in intramolecular dynamics and intermolecular interactions.	Low sensitivity. Out of the millions of incoming photons interacting with molecules, there is only one scattered Raman photon.	[18,25]
Circular dichroism	Measures the difference in adsorption of circularly polarised light. Far-UV CD can determine the absolute and relative contributions of secondary structure types in proteins. Near UV CD can probe tertiary structure content. Can probe changes in protein structure in response to formulation.	A reference protein with known secondary structure is required to fit the experimental data. The quality of the fit also depends on the wavelengths used.	[18,26]
Isothermal titration calorimetry (ITC)	Measures the heat emitted or absorbed during the titration of a protein with a ligand. The amount of heat indicates the proportion of excipient that binds the protein and its associated enthalpy.	ITC can be used to determine the excipient mechanism directly and indirectly. However, no structural information of the protein is given.	[18,27]
Differential scanning calorimetry (DSC)	Routinely used in high-throughput screening of excipients for formulations. Determines the impact of excipients on the thermal stability of the protein, measured as the melting temperature and enthalpy of unfolding.	Useful for identifying excipients that preferentially interact with proteins, or that stabilise through crowding effects. Cannot be used to detect other mechanisms of action. Unable to characterise changes specific to the secondary or tertiary structure of proteins.	[16,28,29,30]
Differential scanning fluorimetry (DSF)	Uses a PCR thermocycler to scan the fluorescence of extrinsic dye-binding to proteins as a function of temperature in microtitre plates, and determine their melting temperatures.	The excitation source of the PCR equipment can potential limit the type extrinsic fluorescence dyes used. Unable to characterise excipient mechanisms of action and can only detect tertiary structure changes.	[16,31,32,33]

**Table 2:** The typical molecular dynamics (MD) simulations timescales that can observe various protein dynamics events.

Protein Dynamics Event	MD Simulation Time Range
Vibrational motions	Femtoseconds (10^−15^) to picoseconds (10^−12^)
Rotational motions	Picoseconds (10^−12^) to nanoseconds (10^−9^)
Loop dynamics	Picoseconds (10^−12^) to milliseconds (10^−3^)
Ligand binding/unbinding	Nanoseconds (10^−9^) to seconds
Protein folding/unfolding	Microseconds (10^−6^) to seconds
Aggregation	Seconds and beyond
